# Computer Literacy Among Students of Zahedan University of Medical Sciences

**DOI:** 10.5539/gjhs.v7n4p136

**Published:** 2014-12-31

**Authors:** Hassan Robabi, Azizollah Arbabisarjou

**Affiliations:** 1 Faculty member, Pregnancy Health Research Center, Zahedan University of Medical Sciences, Zahedan, IR, Iran

**Keywords:** computer literacy, medical sciences, students

## Abstract

**Introduction:**

The need for medical students to be computer literate is vital. With the rapid integration of information technology (IT) in the health care field, equipping students of medical universities withcomputer competencies to effectively use are needed. The purpose of this study was to assess computer literacy (CL) needs of medical sciences students.

**Methods:**

This is descriptive-analytic. The population of the study comprised all students at Zahedan University of Medical Sciences. 385 students from allschools (Medicine, dentistry, paramedics, health, rehabilitation, nursing and midwifery) were selected through randomized- classified sampling. For data collecting, the Lin Tung- Cheng questionnaire was used which it contained 24 items in six sections. The obtained data analyzed by SPSS 15.

**Results:**

The results showed that the 77.1% had personal computer. The total mean of students’ computer literacy around six domains was 141.9±49.5 out of 240. The most familiarity with computers was the ability to it in internet (29.0±11.4) and the lowest was familiarity and using ability of hard ware (17.5±10.6). There was a significant relationship between passing the Computer lesson (P=0.001), passing Computer course (P=0.05) and having personal computer (P=0.001) with the mean of computer literacy.

**Discussion:**

In sum, the medical sciences students’ familiarity with computer literacy was not satisfactory and they had not appropriate familiarity with computer literacy skills. The researchers suggest the officials and in-charges to plan educational program for improving computer literacy skills in medical sciences students.

## 1. Introduction

Computer competencies are essential for success in the business world especially in education. These competencies are a useful tool for students to utilize to integrate them into curricula for all level of students education. Some believe that CL has involved preparation of persons to serve as worthy citizens in their communities and understand how society operates in an information age (Burniske, 2001). Recently, CL has been an issue of educational research in the university. Technological advances necessitate learning, maintaining, and upgrading of computer related knowledge (Hindi, Miller, & Wenger, 2008). CL is an emergent need for medical and paramedical students in the third millennium due to rapidly changing information society. Nowadays, students confront new challenges which vital to their survival in the information age (Ikolo & Okiy, 2012). One of the most applicable for IT and computers is in telemedicine and telehealth. Telehealth is applying the ICT for giving health care remotely. Furthermore, IT-based computer can be used for educating nurses, students, patients or care givers (Arbabisarjou, 2010). In recent years, it grows parallel with the growth of information technology and communication (ICT), using of computer systems, health electronic file and health information system (HIS) are integrated in the life, education and health. There is no agreement on CL; the topic certainly is quite relevant to key stake-holders: students, educators, and business practitioners (Kim & Keith, 1994; Kretovices & MC Cambridge, 1998; Hindi, Miller, & Wegner, 2010). An overview, CL involves conceptual knowledge related to basic terminology (including social, ethical, and global issues) and skills necessary to perform tasks in word processing, databases, spreadsheets, presentation graphics, and basic operating system functions (Hindi, Miller, & Wegner, 2010). Since common technology allied in computer programs (like Microsoft), peer-to-peer programs (like Skype), and social networking programs (like Twitter), we agree with Son, Robb, and Miadji (2011) on what CL is (Murray & Blyth, 2011). Son et al. (2011) wrote” CL is the ability to use computers at an adequate level for creation, communication and collaboration in a literate society. Ballsntine and McCourt et al. measured CL using several sub-dimensions such as “knowledge of general computing”, spreadsheets, databases, email/internet and presentation soft-ware. CL considered as a collection of skills pertaining to the use of basic information and communication technology in an internet-computer based environment as well as the knowledge that relates to the legal and ethical issues and risks of ICT usage. The terms “CL” and “computer knowledge” will be used interchangeably (Poelmans, Truyen, & Desle, 2009). Computer skills are vital for medical practitioners of the future. Health care professional can no longer the application of IT to health care because they are key to E- health (Gour & Srivastavad, 2010). IT has had a positive impact on health care delivery system worldwide, particularly in the areas of disease control, diagnosis, patient management, teaching and learning (Ikolo & Okiy, 2012).

Availability of computers have resulted in our ability to rapidly and effectively,retrieve,analyze, share, and store large volumes of information related to patient care and for teaching-learning process in teaching hospitals (Masood, Khan, & Waheed, 2010) and they noted that computer skills are vital for medical practitioners of the futures. The increasing use of IT in healthcare to posit that healthcare services efficacy and efficiency can be improved through informatics technology and systems and,thus, provide higher–quality healthcare for patients (Tung-Cheng, 2011).

To use technology effectively for the advancement of patient care and the education of the medical students, medical staff must possess a variety of computer skills. Medical field is an information intensive profession. The availability of affordable computers and the advancement of IT have resulted in our ability to rapidly and effectively access, retrieve analysis, share, and store volumes of information pertinent to patient care and for learning process in a teaching hospital (Blen, Miller, & Malyuk, 2000). The final version of the developed CL scale included six constructs (software, hardware, multimedia, networks, information ethics, and information security) and 22 measurement items. Computer experiences has a major positive impact on all our literacy factors well. Exact sciences have a significantly score than students of other branches.

CL is defined as the knowledge and ability to use computers and related technology efficiently, with a range of skills covering levels from elementary use to programming and advanced problem solving (Ikolo & Okiy, 2011). Anuobi (2004) described CL as having a basic understanding of what computer is and how it can be used as resource. Computer skills are vital for medical practitioners of the future. To use technology effectively for the advancement of patient care and the education of the medical students, medical staff must possess a variety of computer skills. Medical field is an information intensive profession. The availability of affordable computers and the advancement of IT have resulted in our ability to rapidly and effectively access, retrieve, analyze, share, and store large volumes of information pertinent to patient care and for learning process in a teaching hospital (Balen, Miller, & Malyuk, 2000).

Lack of Computer skills is an issue which can hold back many of the pedagogical opportunities that students can exploit to assist in their medical sciences teaching (Murray & Blyth, 2011).

Female students have a significant lower CL score than male students (Ikolo & Okiy, 2012). CL for the students should include generic data management, presentation and communication applications as well as search strategies and techniques. For empowerment of medical sciences students in applying computer and IT in medical sciences education and performance of their professional activities in future, awareness of medical sciences students and their interests to learn computer skills in students of Zahedan University of Medical Sciences.

## 2. Review of Literatures

Recently, a number of studies have investigatedthe CL.

Mattheos et al. (2005) investigated CL amongst dental educators and students. The findings showed that the basicCL is a necessary skill for the dentist. There was a positive correlation between gender and competence with computers. The scores for female were very less than males. Saranto and Leino-kilpi (1997) applied the Delphi technique to examine the level of CL necessary in nursing education. They found that basic skills were basic computer operation, word processing skills, spreadsheet and database experience, and internet skills including use E-mail. Ershadisarabi and Bahadini (2005) investigated the CL in medical students. They concluded that the CL score was 56 from total score (100). There was a significant difference between gender and CL. Zarei, Rokhafruz and Diant (2012) carried out a survey on computer literacy in students of general medical students. Their findings showed that the students’ familiarity with CL was not satisfactory. Male students had more CL than female students. Salehi and Hajiabadi (2010) assessed the general CL in employees. They found that general CL was less than moderate.

## 3. Materials and Methods

This is descriptive-analytic. The population of the study comprised all students at Zahedan University of Medical Sciences. 385 students from all schools (Medicine, dentistry, paramedics, health, rehabilitation, nursing and midwifery) were selected through randomized- classified sampling. A questionnaire was used for data collecting which it consisted of two parts. The first part was included 12 items about demographic data and the second part was the Lin Tung- Cheng questionnaire. This questionnaire consisted of six sections with 24 items. The sections were Hardware, Software, Multimedia, Internet, Ethical information and security information. A ten-degree scale was measuring the responses (0 = lack of Skill and 10 = Excellent skill). Furthermore a five –degree Likert scale used too. The scores were classified as 0-2 = Very poor, 3-4 = Poor, 5-6 = Moderate, 7-8 = Good and 9-10 = Excellent). The questionnaire validity was confirmed by expert panel whose comments and suggestions were used to modify the questionnaire. The reliability of the questionnaire was established by Cronbach Alpha and it calculated 0.87. All the survey results were compiled and analyzed with the SPSS-15.

## 4. Findings

The findings revealed that students mean age was 21.9±3.3and 79% were female and 72.5% were resident in collegial residency. The results showed that the 77.1% had personal computer. The total mean of students’ computer literacy around six domains was 141.9±49.5 out of 240. The most familiarity with computers was the ability to it in internet (29.0±11.4) and the lowest was familiarity and using ability of hardware (17.5±10.6). There was a significant relationship between passing the Computer lesson (P=0.001), passing Computer course (P=0.05) and having personal computer (P=0.001) with the mean of computer literacy.

Distribution of fields is illustrated in Table 1.

**Table 1:** Frequencies and percentages of the student’s field

Filed	n	%
Medicine	35	9.1
Dentistry	42	10.9
Nursing	69	17.9
Midwifery	50	13.0
Operation room	62	16.1
Health	76	19.7
Paramedics	51	13.3
Total	385	100

77.1% had personal PC. 80% had passed Computer lesson and 40.5% had participated in a computer workshop or class. The second section of the questionnaire focuses on the types of software uses by the students. Table 2 illustrates how frequency each type of software is applied. The top two uses are: Internet (55.8%) and Email (29.4%). While 75.6 of students have never used SPSS version 21.

**Table 2:** Types of software used

	Never (%)	1-2 times a month (%)	1 times a week (%)	2-3 times a week (%)	Almost every day (%)
Microsoft Word	8.1	48.8	20.8	16.4	**6.0**
PowerPoint	8.8	85.2	19.5	11.4	**2.1**
spss	75.6	16.9	4.2	1.6	**1.8**
Internet	3.4	8.6	10.6	22.3	**55.8**
E mail	11.7	23.9	15.1	20.0	**29.4**
Excel	65.7	23.1	3.6	5.5	**2.1**

The results also showed that respondents have most familiarity and skills with PowerPoint (46%=Excellent) and the respondents the least familiarity and skills with Excel (45.7= very weak). Nearly 18% had excellent knowledge about some hard wares (Mother-Board, memory, and CD-Rom and some of them had not ability to install printer or extra hard wares (40%=very weak). In field of multimedia, the maximum familiarity and skill (33.8%= Excellent) related to Presentation (audiovisual files) and the least familiarity and skill (41%=Very weak) related to a camera film editing. The respondents showed that they have familiarity and skill to search and gathering data through Google and Yahoo (63.1%= Excellent) (Table 3).

**Table 3:** Familiarity and skill about computer

Domain	Measurement item	Very good (%)	Good (%)	Barely Acceptable (%)	Poor (%)	Very Poor (%)
Software (SW)	I understand how to use word processing software such as Microsoft Word.	41	28.8	17.9	4.9	7.3
	I have the ability to use word processing software (such as Microsoft Word) to create each document type.	31.7	24.2	20.8	8.3	7.3
	I have the ability to utilize presentation software (i.e., PowerPoint) to create appropriate presentations.	46	24.9	14	6.5	8.6
	I have the ability to use spreadsheet software (such as Microsoft Excel).	7	10.9	21.8	14.5	45.7

Hardware (HW)	I understand the basic components of a computer system, such as motherboard, memory, and CD-ROM.	17.4	14	22.3	23.1	23.1
	When there are problems with booting the computer, I know simple methods to troubleshoot the problem.	12.5	14.3	26	15.8	31.4
	When purchasing a peripheral device such as a printer, I have the ability to install it myself.	13.8	11.4	18.2	16.6	40
	When purchasing network devices, I have the ability to set up connections and utilize accessory devices by myself.	11.2	12.7	23.1	13.5	39.5

Multimedia (MM)	I know how to use multimedia software (such as Photo Impact) to make appropriate images.	12.2	15.3	23.6	17.1	31.7
	I know how to edit pictures from a digital camera.	12.5	10.9	21.6	17.7	37.4
	I know how to edit videos from a digital video camera.	12.5	8.6	21.8	16.1	41
	I have the ability to play multimedia files (such as .AVI and .MPEG files).	33.8	11.7	13.5	16.6	24.4

Network (NW)	I know how to communicate with others by E-mail.	52.4	13.8	10.4	6.2	17.4
	I know how to communicate with others using communication software such as MSN messenger and Skype.	46	11.9	12.7	8.3	21
	I have the ability to attach a file when I send an E-mail	55.3	8.8	10.9	9.6	15.3
	I have the ability to collect needed information through search engines, such as Google and Yahoo	63.1	13	12.7	2.3	8.8

Information ethic (IE)	I will not disseminate illegal data(such as images, documents, music and software).	58.4	7.3	10.1	8.3	15.3
	I am willing to pay to obtain data legally.	37.1	10.4	21.6	7.3	23.6
	I will not download illegal data such as images, documents, music and software..	44.7	11.7	17.4	4.9	21.3
	I will not install software that is unauthorized.	36.6	13.5	17.1	8.8	23.9

Information security (IS)	I have the ability to install antivirus software myself to prevent virus infections.	27.5	12.2	20.8	9.4	30.1
	I can prevent others from inappropriately accessing my computer information by setting up a password.	43.9	15.6	17.7	5.2	17.7
	I have the ability to set up an appropriate password to reduce the risk of it being deciphered.	50.6	12.2	16.9	5.2	15.1
	I understand that opening unknown files introduces the risk of virus infection.	54.8	12.7	14.5	8.3	9.6

In addition, the findings demonstrated that the total mean of respondents in six- domains was 141.9±49.5 out of 240 scores (Table 4).

**Table 4:** Mean and SD of CL in students

Domain	Average score	Standard deviation
Software (SW)	26.7	8.9
Hardware (HW)	17.5	10.7
Multimedia (MM)	18.0	10.9
Network (NW)	29.0	11.4
Information ethic (IE)	25.9	11.9
Information security (IS)	26.7	11.2
Total	141.9	49.5

There was a significant relationship between passing computer lesson and the mean score of students CL scores (P=0.001). There was, also a significant relationship between passing the Computer course and the mean score of students CL scores (p=0.001). There was not any relationship between gender and academic course with students CL.

## 5. Discussion

CL has been a subject of educational research ever since personal computers were introduced to the classroom, either as teaching aids or as tools for self-study. This study has investigated the CL of medical sciences students.

77.1% of respondent had personal PC while in Ershadsarbi (65.6%) had PC(2005). The total mean of students’ literacy around six domains was 141.9±49.5 out of 240 scores, while Ershadisarabi’s study showed that the students got 56% of score. The results revealed that students’ familiarity and skill about Internet and WEB are fair. This result is consistent with Samuel et al. (2004) findings. They found that the highest performance of students was with Email and Internet. In another study in Japan, 43% of students have moderate literacy about Internet and 28% of respondents had good literacy (Murray & Andrew, 2011). Skill and familiarity of students about Microsoft Office such as Excel wasless while they have good familiarity and skill with PowerPoint and Words. The reason may be that they used these software for doing their home works and projects. This findings are consistent with Zarei et al. (2012) and Karami, Khajeh (2007). In Masood and Waheed (2010) study 69% of students had the ability to use power point for presentation, while 47% had the ability to use Excel program.

The results showed that the least score was related to familiarity and skill of hard wares and multimedia that this finding is consistent with findings of Lotfnejadafshar et al. (2007). They concluded that few students had familiarity and skills about hard wares and multimedia. Poelmans et al. (2009) found that 47% of respondents gained 4 outof 6 score about using the Multimedia. Massod and Waheed (2010) study showed that 62% of students had Multimedia skills.

Education and training are necessitated to promote students skills and knowledge about using computer. There was a significant relationship between passing computer lesson and the mean score of students CL scores (P=0.001). There was, also a significant relationship between passing the computer course and the mean score of students CL scores (p=0.001). Inversely, Lotfnejadafshar et al. (2007) found that there was no relationship between them (p=0.399). Therefore, official training course suggested to enable students to use computer to carry out their take homes, project, researches.

There was not any relationship between genders with students CL. The investigation of Waasserman and Richmon_Abbot (2005) showed that the male and female have equal access use of Computers. While Ershadsarabi et al. (2005) and Matz et al. (2005), Ikolo and Okiy (2012) found that there was gender differences in the CLof students. This can be as a result of the fact that the make students seemed very interested in owning their own computers. This findings is consistent with the results of Ershadsarabi et al. (2005), Matz et al. (2005) and Zarei et al. (2012). They concluded that male students CL were higher than females (p<0.001).

Zarei et al. (2012) concluded that the pharmacist students had the most CL scores and the dentistry students had the least CL score among medical sciences students, while the findings ofthis studycleared that there was not any relationship between academic courses with students CL. The rapidly expanding use of computer medicine and biomedicine has changed the medical education methods, whatever CL, the ability to use computer, is an essential skill for medical sciences students. Knowing the CL level of students and the level of their interest for learning IT make students to use computers and IT better in daily and professional lives. As extending access to IT and it expected more developments in ICT systems (Arbabisarjou, 2010), there is need to develop computer skills and CL. The results showed that the students CL was not really fair, hence it suggested to design and plan for classes and workshop to improve CL skills in the universities of medical sciences.
